# Maze Control Training on Kinesthetic Awareness in Patients with Stroke: A Randomized Controlled Trial

**DOI:** 10.1155/2022/5063492

**Published:** 2022-02-24

**Authors:** Hanan Hosny M. Battesha, Amir N. Wadee, Marian M. Shafeek, Ahmed M. Tawfick, Hoda M. Ibrahim

**Affiliations:** ^1^Department of Physical Therapy for Neuromuscular Disorders and Its Surgery, Faculty of Physical Therapy, Modern University for Technology and Information, Cairo, Egypt; ^2^Department of Physical Therapy for Basic Sciences, Faculty of Physical Therapy, Cairo University, Cairo, Egypt; ^3^Department of Physical Therapy for Pediatrics and Its Surgery, Faculty of Physical Therapy, Modern University for Technology and Information, Cairo, Egypt; ^4^Department of Physical Therapy for Internal Medicine and Elderly, Faculty of Physical Therapy, Modern University for Technology and Information, Cairo, Egypt; ^5^Department of Physical Therapy for Musculoskeletal Disorders and Its Surgery, Faculty of Physical Therapy, Modern University for Technology and Information, Cairo, Egypt

## Abstract

**Objective:**

To determine the influence of adding maze control training to the selected conventional physical therapy on kinesthetic awareness in patients with chronic stroke.

**Methods:**

Thirty adult patients of both genders with chronic cerebral stroke were assigned to control and experimental groups randomly: the control group (A) received the selected conventional physical therapy rehabilitation program, while the experimental group (B) received the same program as group A in addition to the maze control training. Measurements for sway index, risk of fall, and knee proprioception before and after 8 weeks of treatment (24 sessions; three times per week).

**Results:**

There were significant decreases of both sway index and risk of fall in both groups (*p* ≤ 0.001 in all measures), significant improvements of the knee proprioception in 30° and 75° in the experimental group (*p* value = 0.016 and ≤0.001, respectively). The in-between groups' comparison showed significant differences corresponding to both the sway index and risk of fall (*p* ≤ 0.001), and a significant difference in 75° (*p* ≤ 0.001).

**Conclusion:**

Adding maze control training to the selected conventional physical therapy improved the kinesthetic awareness in patients with chronic stroke.

## 1. Introduction

Most patients with chronic stroke have postural instability in both static and dynamic positions resulting in difficulty in walking activities and a high tendency of falling, which lead to difficulties in activities of daily living [1]. Therefore, it is important to add proprioceptive training to the rehabilitation program for such cases [2]. Kinesthetic awareness means the direct focus on some specific sensory aspects of the body to detect the outer or inner environment to keep the body's position and movement [3].

The motor control system should be aware of the static and dynamic joint status to represent the complex mechanical connections inside the segments of the musculoskeletal system. Kinesthetic awareness is responsible for providing the motor system with such data [4]. Kinesthetic awareness disorder could extremely affect stroke patient abilities in postural control that increase postural sway and risk of fall, so proprioceptive training is effective in patients with chronic stroke [5].

The risk of fall is common after strokes; patients have an estimated 14% risk of falling that leads to activity limitation, increased dependence, impaired mobility, and reduced body balance, so evaluation of the risk of fall is very important for those patients [6].

For maintaining the body's postural stability, different sensory inputs should be accurate including visual, vestibular, and somatosensory sense [7]. Research reports damage to such sensory system with stroke patients with a high rate of proprioception affection, which affects both static and dynamic postural stability. Therefore, evaluation by the clinical test of sensory interaction and balance (CTSIB) should be considered for both aspects of static and dynamic postural control by the ability to control the tilting angle of the Biodex platform [8].

Multisensor examination was considered a valid and reliable tool to measure dynamic balance and assess the risk of falling before and after neurorehabilitation in patients with subacute stroke [9]. Visual-spatial training was considered a neurorehabilitation to enhance posture stability in patients with subacute stroke [10].

Maze control training was described in the manual of many Biodex balance system machines (BBS) to improve posture stability, trunk control, and static and dynamic balance. It was defined as the ability of the patient to follow a reproducible pattern of movement throughout visual biofeedback on BBS in both static and dynamic environments [11].

So, was there an influence of maze control training on kinesthetic awareness in patients with chronic stroke? The study's purpose was to assess the influence of maze control training on kinesthetic awareness in patients with chronic stroke, and it was hypothesized that it had no influence.

## 2. Methods

### 2.1. Study Design

A randomized control trial pre- and postexperimental design was carried out from June to December 2019 at the rehabilitation center for the neurological patients at the Faculty of Physical Therapy, Modern University for Technology and Information, Cairo, Egypt.

### 2.2. Participants

Thirty referred patients with right chronic stroke (after six months of injury), their ages were ranged from 45 to 65 years, from both genders (21 males and 9 females). The main inclusion criteria included the following: All patients with middle cerebral artery stroke leading to right cerebral hemisphere infarction were confirmed by brain computed tomography scan (CT) at the beginning of injury and two weeks after. All patients have left hemiplegia of the nondominant side confirmed by the Right Hand/Left Hand Hit-the-Dot test [12]. The motor deficit of Brunnstrom was stage IV [13] which means mild impairment of posture stability, and all patients can stand and walk even using an assistive device. Also, their blood glucose level and blood pressure should be controlled level (hemoglobin A1C value ≥ 6.5‐7% and blood pressure < 80‐90/120-140 mmHg).

The exclusion criteria included any patients with a previous cerebrovascular attack, proprioceptive impairment because of peripheral vascular disease or neuropathy, visual disturbances, balance disturbances rather than stroke (e.g., ear problems, labyrinthitis, or diabetic neuropathy), cardiac problems, cognition problems (Mini-Mental State Examination, cutoff score 24) [14], gross motor deficits (limb apraxia), history of pedal ulcer in the lower limb, amputation, advanced arthritis, osteoporosis, malunion fractures, obesity (body mass index BMI over 30 kg/m^2^), and nerve root compression (radiculopathy).

### 2.3. Randomization

**Figure 1:**
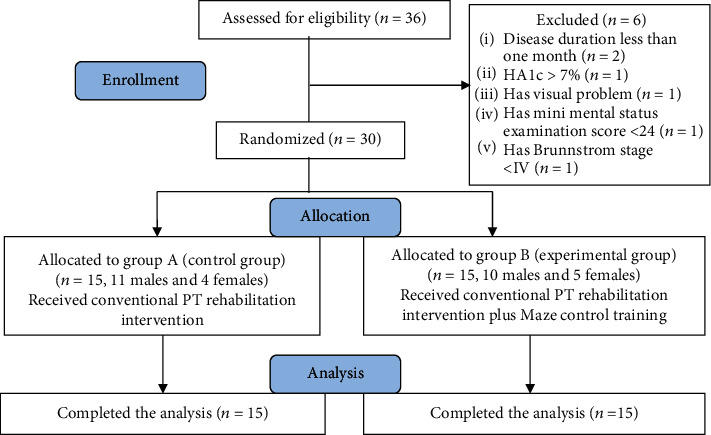
Flow chart of the participants.

Patient randomization into two equal groups (control and experimental) 15 for each was done using the closed envelope. At first, study procedures were described for all patients, and they have signed the consent form for their participation (Figure 1).

### 2.4. Ethical Approval

The study protocol was approved by the Research Ethical Committee, Faculty of Physical Therapy, Cairo University, Egypt (No: P.T.REC/012/002306). Also, the study was registered on Pan African Clinical Trial Registration (PACTR 201904904847417).

### 2.5. The Sample Size Power Analysis

Overall, thirty participants satisfying the inclusion conditions were involved in the study, using G∗Power statistical software, a sample size calculation was done (version 3.0.10), and it was discovered that the suitable sample size was *n* = 30.

### 2.6. Outcomes

Sway index was the primary outcome in patients with chronic stroke, while secondary outcomes were the risk of falling and knee proprioception.

#### 2.6.1. Biodex Balance System (BBS)

**Figure 2:**
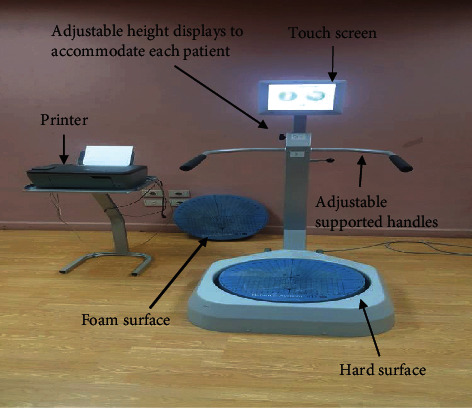
Biodex balance system.

Biodex Medical Systems Inc., Brookhaven R&D plaza, 20 Ramsey Road Box 702, Shirley, New York 11967-0702 (Figure 2). The BBS is a reliable and valid assessment and treatment device for body balance with twelve levels of stability [15]. The device consists of a rounded foot platform that is allowing movement in all directions, adaptable handrails, an alterable colored screen, and connected to a printer.

#### 2.6.2. Isokinetic Dynamometer

The Biodex System 3 Isokinetic Dynamometer (BID) was used to measure proprioception (joint position sense) of the knee joint. The BID consists of a dynamometer, a chair, and a control panel controlled by a computer to collect data and show the results of the test on its screen and restore data; it also controls the motion of the dynamometer. It provides objective, reliable, and valid proprioception testing for joints of the upper limb, lower limb, and trunk. It provides an objective method for assessment of the proprioception of different joints of the body [16]. A typewriter-style keyboard can be used to enter all information about the patient's data into the processor [17].

### 2.7. Testing Procedure

Calibration was performed before the beginning of the testing procedures. All of the participants had their sway index, risk of falling, and knee proprioceptions measured before and after treatment. Before the evaluation procedures, they were given an explanation session to familiarize themselves with the various test proceedings. All tests were performed in the same place under the same conditions by the principal investigator.

#### 2.7.1. Sway Index

The CTSIB is a standard test for balance assessment on different surfaces. This test was designed to assess the patient's ability to integrate sensory information to maintain body equilibrium. The result of the CTSIB test is the sway index, which indicates the mean absolute deviation of the center of gravity; the more sway index means loss of posture stability. All participants stand on the platform of BBS bare feet at an angle between 25° and 30° and adjusted the stability level at the 8^th^ level for 10 seconds (test period) with three trials. The four conditions in CTSIB were selected, firstly on a hard surface with open eyes, secondly on a hard surface with closed eyes, thirdly on a foam surface with open eyes, and finally on a foam surface with closed eyes. Ask all participants to maintain the posture stability as much as they can with no posture sway [18].

#### 2.7.2. Risk of Fall

The measurement of fall of risk test on BBS was done by asking the participant to maintain the static erect posture on a dynamic platform for 20 seconds with three trials [19].

#### 2.7.3. Knee Proprioception

The participant was seated on the chair of the dynamometer with flexion 90° of both lower limbs and maintained this position by straps around the thigh and trunk. Ask the participant to extend his/her knee to selected target three angles (15°, 30°, and 75°) and hold the knee position at each angle for 10 seconds to stimulate different joint proprioception [20].

### 2.8. Interventions

#### 2.8.1. Group A (Control Group)

The control group received the selected conventional physical therapy rehabilitation program only for eight weeks (24 sessions; three times per week). It included the following:

*(1) Conventional Gait Training*. The participant has performed the leaning forward from sitting and standing positions, sitting from chair to stand, heel to toe stand, stand on tiptoes, walking in a figure of eight, and up and down steeper for 10 minutes followed by a rest period for 3 minutes [21].

*(2) Proprioceptive Neuromuscular Facilitation (PNF) for the Lower Limb*. The specific technique of PNF as an agonist to facilitate agonist (repeated contraction) was performed. Flexion-abduction-internal rotation and flexion-adduction-external rotation patterns were used for 10 minutes followed by a rest period for 3 minutes [22].

*(3) Functional Activities of Daily Living Retraining*. Regaining the ability to perform the activities of daily living with focusing on retraining on transferring activity especially with using mobility aids and also grasp and release activity for 10 minutes followed by a rest period for 4 minutes [23].

*(4) Selected Proprioceptive Training*. Patients had received the different phases of proprioceptive training as static standing on one leg, standing on balance board, squat position, and walking in a straight line on a hard surface then on foam surface for 20 minutes, all proprioceptive exercises done firstly with open eyes then with closed eyes [7].

#### 2.8.2. Group B (Experimental Group)

Participants were receiving the selected conventional physical therapy rehabilitation program in addition to the maze control training for 20 minutes (instead of the selected proprioceptive training). The treatment duration was for eight weeks (24 sessions; three times per week).

*(1) The Maze Control Training*. All participants were standing on a platform of BBS with bare feet at the angle between 25° and 30° degrees and supported both hands on the handrails. The selected level of training is level eight of stability. Once the platform had a motion, ask the participant to follow a set of targets through a maze. The participant moves the cursor to each target as it blinks and did not let the cursor touch a wall of the maze. The percentage of net good hits divided by the total target hits determines the score, and any hits outside the boundary are subtracted from the total target hits.

### 2.9. Statistical Analysis

IBM SPSS version 22 (IBM Corporation, USA) was used in carrying out all statistics. The sway index was chosen as the primary outcome measure. The sample size calculation required at least 12 patients in each group. The total sample reached 30 patients to allow 20% as a dropout. All data were homogenous (Levene's homogeneity test). All data were normally distributed (Shapiro-Wilk normality test), so a parametric test was used (MANOVA). The results were considered statistically significant when the *p* values were less than 0.05.

## 3. Results

### 3.1. Baseline Characteristics

The age and BMI of patients in both the control and experimental groups were homogeneous (*p* value = 0.793 and 0.434, respectively). The numbers of males to females in both control and experimental groups were 11 : 4 and 10 : 5, respectively (Table 1).

Mixed MANOVA was conducted to investigate the effect of treatment on sway index, risk of fall, and knee proprioception. There was a significant effect of treatment, the interaction effect of treatment and time, the interaction effect of treatment and groups, and the interaction effect of treatment, groups, and time (*p* ≤ 0.001, and partial eta squared = 0.991, 0.55, 0.394, and 0.394, respectively).

### 3.2. Sway Index

The within-group comparisons showed statistically significant decreases in both control and experimental groups (*p* ≤ 0.001). The in-between group comparison showed statistically insignificant decreases in the pretest while highly significant decreases in the posttest in favor of group B (*p* value = 0.782, 0.616, 0.571, 0.739, and ≤0.001^∗^ for all measures, respectively) (Table 2).

### 3.3. Risk of Fall

The within-group comparisons showed statistically significant decreases in both control and experimental groups (*p* ≤ 0.001). The in-between group comparison showed a statistically insignificant decrease in the pretest while a significant decrease in posttest (*p* value = 0.805 and ≤0.001, respectively) (Table 3).

### 3.4. Knee Proprioception

The within-group comparisons showed statistically insignificant decreases in the control group (*p* value = 0.373, 0.641, and 0.618, respectively) while an insignificant decrease in 15° and significant decreases in both 30° and 75° in the experimental group (*p* value = 0.098, 0.016, and ≤0.001, respectively). The in-between group comparison showed statistically insignificant decreases in both the pretest and posttest measurements except in 75° (*p* value = 0.802, 1, 0.918, 0.395, 0.058, and ≤0.001, respectively) (Table 4).

## 4. Discussion

The current study was conducted to assess the effect of maze control training on kinesthetic awareness in patients with chronic stroke. The within-group comparisons showed statistically significant decreases of both sway index and risk of fall in both groups (*p* ≤ 0.001 in all measures); these findings corroborate with others who showed a statistically significant improvement in the balance following proprioceptive exercises for patients in the chronic recovery stage of stroke [24].

Regarding the knee joint proprioception, there were nonsignificant improvements in the control group in all degrees, while significant improvements in 30° and 75° in the experimental group (*p* value = 0.373, 0.641, 0.618, 0.098, 0.016, and ≤0.001, respectively). That confirmed the favor of maze control training when added to the conventional physical therapy rehabilitation program and add more evidence to add specific exercises to the traditional program for aiming to enhance the regain of static and dynamic balance abilities for a stroke patient [25].

Within the limitations of the concurrent study, the in-between group comparison showed significant differences corresponding to both the sway index and risk of fall (*p* ≤ 0.001 for both measures). The previous study has supported the use of nontraditional balance training exercises to improve kinesthetic awareness for individuals with chronic stroke, who recommended future high-quality, controlled studies to investigate the effects of different balance training for those patients [26]. This improvement may be explained by several activity-focused approaches to balance challenges that were efficient [27] and add proof that the maze control program is an efficacious rehabilitation program for improving balance for patients with chronic stroke. Also, it could be similar to the effect of virtual reality (VR) training in balance recovery as both maze control and VR act on visual feedback training to improve posture stability, which is less clear in patients with chronic stroke, and the previous researches stated the need for further research to investigate its optimum training intensity and frequency to achieve the desired outcome from visual feedback training [28, 29].

As a consequence, the in-between group comparison results in this study also demonstrated nonsignificant improvement in knee proprioception in both 15° and 30°, while significant improvement in 75° (*p* = 0.395, 0.058, and ≤0.001, respectively). This confirmed the use of maze control training, as visual biofeedback, in rehabilitation seems to be useful to improve kinesthetic awareness. Muscle receptors are acting a necessary function at the midrange of motion (15° and 30°) while ligament receptors are functioning near the end of the knee range (75°) [30] that describes the results of this study. Therefore, the physiotherapy plan should be directed to guide the patient into the most suitable motor strategies; also, the flexibility of the software is highly recommended to be more individualized for each patient's condition [31].

According to the obtained improvements in sway index, risk of fall, and proprioception in the experimental group, we could recommend adding the maze control training to a conventional physical therapy rehabilitation program in patients with chronic stroke as it can significantly improve kinesthetic awareness and, as a consequence, it will have an impact on patients' posture control and performance motor function and reduce the risk of fall [32].

On the same line, another study found that treadmill training (TT) recognized moderate evidence in balance rehabilitation of patients with stroke. The intensity of the TT program was a most important factor than specificity, and it adds improvement not only in gait abilities but also postural control and balance abilities [33]. So, future studies were recommended to compare maze control training versus TT and impact on balance and transfer abilities.

The possible explanation for improvement that occurred in the experimental group is that the maze control training may enhance the peripheral somatosensory stimulation (PSS) that improves postural control of the stroke patient as a result of enhancement of sensory input and postural contribution for the affected lower limb. PSS was an effective therapy for improving postural stability in a chronic stroke patient; it has immediate and long-term effects [34].

Previous review studies reported limited evidence for some interventions for stroke patients including repetitive task training, exercise therapy, care-mediated exercise, physical fitness training, use of unstable support surfaces, and virtual therapy that may be effective in balance recovery after stroke. For confirming this finding, further research is recommended [8, 35].

### 4.1. Limitations

The authors are aware of the study's limitations. This examination was constrained by diminished patient's capacity to complete with treatment procedures, as patients were all of a sudden feeling migraine, inconvenience concentrating, blurred vision, and fatigue (powerless and tired inclination). It was the psychophysiological factors for patients at the time of examination and training, which was assumed to be the same for patients all over the study. We also could not capture the lower limbs' muscle activity during balance training, because of not having an electromyography (EMG) device. If we had designed a weight shift training test on BSS, by an EMG device, we would have had a better justification for the muscles' activity.

## 5. Conclusion

Adding the maze control training program to the conventional physical therapy rehabilitation program could be more useful and effective for improving kinesthetic awareness than the conventional physical therapy rehabilitation program alone in patients with chronic stroke. Further studies are needed to demonstrate its effective superiority over conventional physical therapy training in more sample sizes and for a longer rehabilitation period.

## Figures and Tables

**Table 1:** Baseline characteristics of the subjects in both groups.

Demographic data		Control group	Experimental group	*T* value	*p* value
Age (years)		61.8 ± 1.12	62.27 ± 1.31	0.27	0.79
BMI (kg/m^2^)		28.91 ± 0.75	29.77 ± 0.78	0.79	0.44
				Chi-square value	*p* value
Gender	Males	11 (73.33%)	10 (66.67%)	0.16	0.69
	Females	4 (26.67%)	5 (33.33%)		

**Table 2:** Sway index in open eyes-firm surface, closed eyes-firm surface, open eyes-foam surface, and closed eyes-foam surface within and in-between groups.

Sway index		Control group	Experimental group	In-between group comparison *p* value
Open eyes-firm surface	Pretest	1.93 ± 0.13	1.94 ± 0.14	0.782
	Posttest	1.14 ± 0.16	1.12 ± 0.29	≤0.001^∗^
	Within-group comparison *p* value	≤0.001^∗^	≤0.001^∗^	
Closed eyes-firm surface	Pretest	2.44 ± 0.17	2.42 ± 0.12	0.661
	Posttest	1.96 ± 0.28	1.29 ± 0.11	≤0.001^∗^
	Within-group comparison *p* value	≤0.001^∗^	≤0.001^∗^	
Open eyes-foam surface	Pretest	2.67 ± 0.37	2.62 ± 0.22	0.571
	Posttest	1.82 ± 0.16	1.34 ± 0.07	≤0.001^∗^
	Within-group comparison *p* value	≤0.001^∗^	≤0.001^∗^	
Closed eyes-foam surface	Pretest	4.81 ± 0.20	4.79 ± 0.2	0.739
	Posttest	3.99 ± 0.22	3.22 ± 0.28	≤0.001^∗^
	Within-group comparison *p* value	≤0.001^∗^	≤0.001^∗^	

**Table 3:** Risk of fall within and in-between groups.

Risk of fall	Control group	Experimental group	*p* value in-between groups
Pretest	3.10 ± 0.13	3.11 ± 0.27	0.805
Posttest	2.35 ± 0.19	1.48 ± 0.15	≤0.001^∗^
*p* value within groups	≤0.001^∗^	≤0.001^∗^	

**Table 4:** Knee proprioception in 15°, 30°, and 75° within and in-between groups.

Knee proprioception		Control group	Experimental group	*p* value In-between groups
Proprioception in 15°	Pretest	6.48 ± 1.97	6.33 ± 1.68	0.802
	Posttest	5.95 ± 1.80	5.52 ± 1.4	0.395
	*p* value within groups	0.373	0.098	
Proprioception in 30°	Pretest	7.14 ± 2.43	7.14 ± 2.15	1
	Posttest	6.81 ± 2.16	5.67 ± 1.59	0.058
	*p* value within groups	0.641	0.016^∗^	
Proprioception in 75°	Pretest	7.55 ± 1.60	7.60 ± 1.38	0.918
	Posttest	7.30 ± 1.72	5.81 ± 0.87	≤0.001^∗^
	*p* value within groups	0.618	≤0.001^∗^	

## Data Availability

The data used to support the findings of this study are available from the corresponding author upon request.
